# Transgressive Hybrids as Hopeful Monsters

**DOI:** 10.1007/s11692-012-9209-0

**Published:** 2012-11-20

**Authors:** Dylan R. Dittrich-Reed, Benjamin M. Fitzpatrick

**Affiliations:** University of Tennessee, 569 Dabney Hall, Knoxville, TN 37996 USA

**Keywords:** Hopeful monster, Transgressive segregation, Hybrid speciation, Phenotypic novelty

## Abstract

The origin of novelty is a critical subject for evolutionary biologists. Early geneticists speculated about the sudden appearance of new species via special macromutations, epitomized by Goldschmidt’s infamous “hopeful monster”. Although these ideas were easily dismissed by the insights of the Modern Synthesis, a lingering fascination with the possibility of sudden, dramatic change has persisted. Recent work on hybridization and gene exchange suggests an underappreciated mechanism for the sudden appearance of evolutionary novelty that is entirely consistent with the principles of modern population genetics. Genetic recombination in hybrids can produce transgressive phenotypes, “monstrous” phenotypes beyond the range of parental populations. Transgressive phenotypes can be products of epistatic interactions or additive effects of multiple recombined loci. We compare several epistatic and additive models of transgressive segregation in hybrids and find that they are special cases of a general, classic quantitative genetic model. The Dobzhansky-Muller model predicts “hopeless” monsters, sterile and inviable transgressive phenotypes. The Bateson model predicts “hopeful” monsters with fitness greater than either parental population. The complementation model predicts both. Transgressive segregation after hybridization can rapidly produce novel phenotypes by recombining multiple loci simultaneously. Admixed populations will also produce many similar recombinant phenotypes at the same time, increasing the probability that recombinant “hopeful monsters” will establish true-breeding evolutionary lineages. Recombination is not the only (or even most common) process generating evolutionary novelty, but might be the most credible mechanism for sudden appearance of new forms.

## Revival of the Hopeful Monster

A major task for evolutionary biology has been to develop and test theories for the origin of novelty that are consistent with the fundamental genetic principles of gradual populational change. Novelty, however, is a loaded term with many different definitions that include or exclude a variety of morphological characters (Brigandt and Love 2012). Following Pigliucci (2008), we prefer a more inclusive definition of evolutionary novelty: new traits, or novel combinations of traits within a lineage that perform a new ecological function and may result in the establishment of new evolutionary lineages. More narrowly focused definitions might be desirable for some purposes (Muller and Wagner 1991; Wagner and Lynch 2010). However, our goal in this essay is to elaborate one mechanism for the sudden origin and evolutionary success of new variants that applies just as well to exceptional size and shape, new color patterns, use of new habitats, and new exons.

Some theorists have invoked special phenomena such as genome-wide “macromutations” (Goldschmidt 1940) or “genetic revolutions” (Mayr 1954) to get around perceived difficulties with the emergence of profound change as the accumulation of subtle changes by the conventional dynamics of mutation, gene flow, drift and selection. However, modern evolutionary theory and empirical research in genetics have consistently reaffirmed the ability of conventional population genetics to explain the origin of new species and phenotypes, and simultaneously exposed flaws in the alternatives (Charlesworth et al. 1982; Lynch 2007). For example, Goldschmidt (1933, 1940) proposed that a novel phenotype (such as insect wings, a character associated with higher level taxonomy) must first arise as an instantaneous product of a single “macromutation” or “systemic mutation”. Individuals bearing such macromutations were characterized as “hopeful monsters” by Goldschmidt (1933, 1940) to emphasize that their appearance is neither purposeful nor gradual, and their prospects for success are a matter of luck. A hopeful monster is an individual phenotypically discontinuous from the range of phenotypes of its population, and whose hopes of establishing a new lineage lie in finding a novel niche for which its monstrosity happens to be preadapted. Such a mechanism of speciation was criticized early for being so improbable as to “overtax one’s credulity” (Dobzhansky 1937, p. 53) because of the rarity of the initial mutation of large effect, and the resulting improbability of finding an equally monstrous mate (Dobzhansky 1937).

**Fig. 1:**
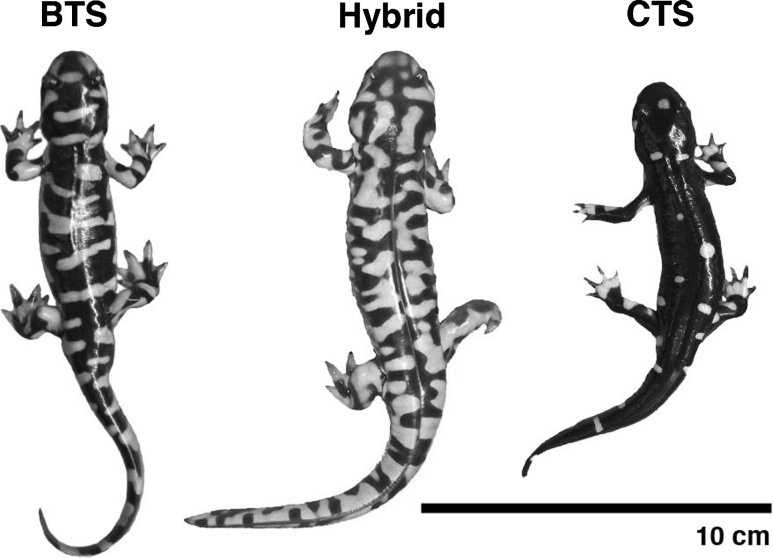
Recently metamorphosed juvenile tiger salamanders representative of *Ambystoma mavortium* (BTS), *A. californiense* (CTS) and transgressive later generation hybrid. The late generation hybrid has both a transgressive coloration and body size (mass and snout-vent length) beyond the range of parental populations

Recent empirical and theoretical research on hybrid speciation might have revived the hopeful monster in a new, more credible form (Mallet 2007). Recombination of parental chromosomes in the F_2_ and later generations during hybridization can generate genotypes that express phenotypes outside the normal range of variation observed in either parental gene pool, a phenomenon termed “transgressive segregation” (Fig. 1; Rieseberg et al. 1999, 2003; Rosenthal et al. 2005; Johnson et al. 2010; Parsons et al. 2011). Often, transgressive hybrids have higher fitness in novel environments, increasing the likelihood of divergence from parental populations (Arnold and Hodges 1995; Buerkle et al. 2000; Gompert et al. 2006; Karrenberg et al. 2007; Rieseberg et al. 2007; Shahid et al. 2008; Abbott et al. 2010; Fitzpatrick et al. 2010). A few examples of new phenotypes inferred to arise from hybridization include (see Arnold 1997; Arnold 2006; Stelkens and Seehausen 2009 for more exhaustive reviews): extreme size of tiger x lion F_1_ hybrids (Gray 1954); unique shapes and colors of hybrid orchids (Rolfe and Hurst 1909); ability of recombinant sunflowers to thrive in extreme habits (Lexer et al. 2003; Rieseberg et al. 2003, 2007); specialization on a novel host plant in lonicera flies (Schwarz et al. 2005); and expression of novel gene transcripts (including new exons) via alternative splicing in hybrid poplars (Scascitelli et al. 2010). Not all specific examples are relevant in nature, and not all would qualify as “evolutionary novelty” under certain definitions (Muller and Wagner 1991; Pigliucci 2008; Wagner and Lynch 2010), but this small selection of cases serves to illustrate sudden appearance of profound differences between parents and hybrid offspring reminiscent of Goldschmidt’s hopeful monsters.

**Fig. 2:**
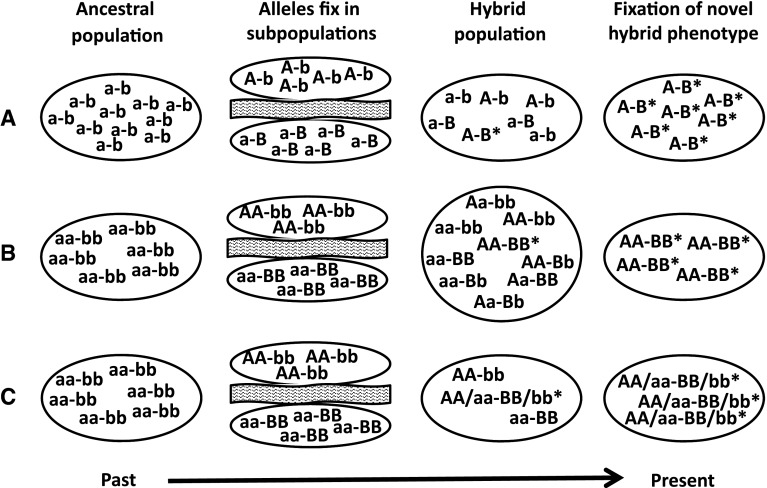
Genetically explicit versions of Bateson’s model. **a** The haploid case, **b** the diploid case, **c** allopolyploidy. Genotypes with *asterisks* are novel recombinant, true-breeding genotypes

Arnold and colleagues have promoted the importance of transgressive segregation as the “evolutionary novelty” model of hybridization (Arnold 1997; Arnold et al. 1999; Arnold et al. 2012). Mallet (2007) even referred to transgressive hybrids as hopeful monsters, and P. Bateson (1984, 2002) proposed a simple model for the sudden appearance and successful spread of a novel phenotype via hybridization as a mechanism of saltational evolution. We expand and make genetically explicit the haploid, diploid and polyploid cases of his model (Fig. 2). It is related to other models of transgressive segregation (Rieseberg et al. 2003) and hybrid fitness (Dobzhansky 1937; Muller 1942; Turelli and Orr 2000). All are special cases of a general multilocus model (Fitzpatrick 2008) which can give rise to the evolution of novelty or discontinuity as the cumulative or combined outcome of conventional population genetic change. Indeed, recombination has always been recognized as an important source of variation (Mendel 1866); whether such variation is perceived as profound or “monstrous” is a matter of degree rather than kind.

## The Bateson Model

**Fig. 3:**
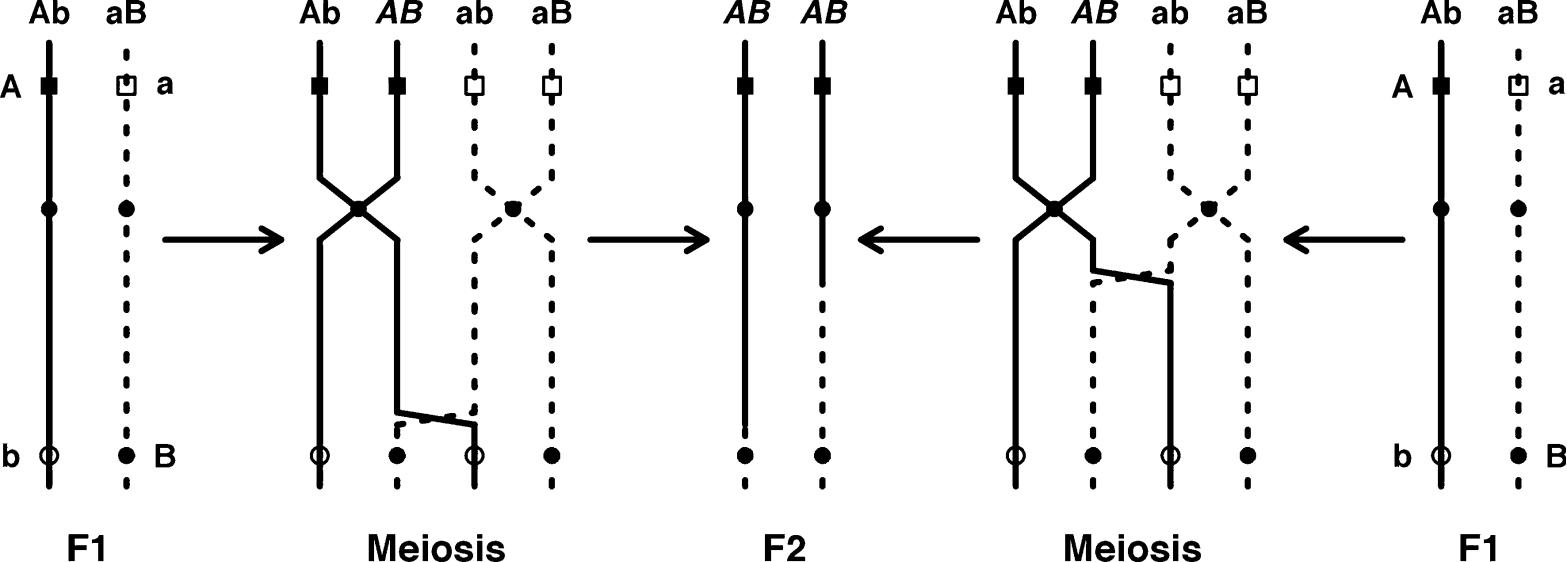
A schematic representation of the process by which two fixed allelic differences (A and B) at unlinked loci might recombine during meiosis in two F_1_ hybrids to create a novel homozygous genotype (AABB) in the F_2_ hybrid. *Solid* and *dashed* chromosome patterns are indicative of population ancestry. Note that the two novel recombinant chromosomes in the F_2_ are the result of independent recombinational events

Bateson’s (1984, 2002) proposal for how recombination can generate sudden change is a straightforward narrative. Two different mutations (*A* and *B*) appear and become fixed in different populations with similar phenotypes (circles in his diagram). When the populations merge, recombinant individuals with both *A* and *B* express a new phenotype (diamonds in his diagram), which is more successful and becomes fixed. Aside from “mutation”, Bateson did not use genetically explicit vocabulary, but his diagram suggests a haploid genome, with mutations *A* and *B* occurring in different loci such that recombination can place them together in the same individual. We show a version of Bateson’s model with explicit haploid, diploid, and allopolyploid cases in Fig. 2. The key feature is that the new phenotype depends on the interaction between alleles *A* and *B* at different loci. If both *A* and *B* alleles are common in the admixed population, the new phenotype will be expressed by a large number of individuals who can interbreed with each other, rather than a single mutant monster with no prospect for a mate. Moreover, even if interactions at other loci render some hybrids (even F_1_ hybrids) partly or mostly sterile, recombination could produce transgressive hybrids with restored fertility in the F_2_ and later generations (Fig. 3).

## The General Model

Bateson (2002) went on to note that his idea had “points of similarity” with the Dobzhansky-Muller model of hybrid dysfunction (Dobzhansky 1937; Muller 1942; Turelli and Orr 2000) and the earlier verbal model of W. Bateson (1909). In fact, the explicit diploid version of Bateson’s model differs from the Dobzhansky-Muller model only in the sign of the interaction: The Bateson model supposes the interaction between *A* and *B* increases fitness, while the Dobzhansky-Muller model specifies a decrease in fitness of recombinant hybrids (Table 1A, B). Both models describe gene interaction (epistasis) causing a hybrid phenotype to fall outside the range for either parental population. That is, they are special cases of transgressive segregation.

**Table 1:** Diploid, two-locus models for hybrid phenotypes

	aa	Aa	AA
(A) Epistatic hybrid dysfunction			
bb	1	1	1
Bb	1	1 − *h*_0_	1 − *h*_1_
BB	1	1 − *h*_1_	1 − *h*_2_
(B) Epistatic hybrid vigor			
bb	1	1	1
Bb	1	1 + s_0_	1 + s_1_
BB	1	1 + s_1_	1 + s_2_
(C) Additive complementation			
bb	1 − *2x*	1 − *x*	1
Bb	1 − *x*	1	1 + *x*
BB	1	1 + *x*	1 + 2*x*

In each case, parental genotypes are AAbb aaBB. Epistatic hybrid dysfunction (A: the Dobzhansky-Muller model) and epistatic hybrid vigor (B: the Bateson model) differ only in whether effects are assumed to be deleterious or beneficial. The additive complementation model (C) shows how recombinants can be phenotypically extreme relative to parentals (AAbb and aaBB) even without gene interaction (each A or B allele contributes an amount *x* to the phenotypic value, regardless of the other locus). All can be written as special cases of a general quantitative genetic model (Hill 1984; Lynch and Walsh 1997; Fitzpatrick 2008)

Transgressive segregation can also be caused by strictly additive effects of multiple genes (Table 1C; Nilsson-Ehle 1911; Grant 1975). This is the genetic model favored by Rieseberg et al. (2003) because in QTL studies of transgressive hybridization in plants, additive effects are detected more often than epistatic or dominance interactions (Rieseberg et al. 1999). Strictly additive and strictly epistatic models are special cases of the general quantitative genetic model allowing phenotypes to be affected by additive, dominance, and epistatic effects (Hill 1984; Lynch and Walsh 1997; Fitzpatrick 2008). Extending these basic ideas to many loci and multivariate phenotypes leads to the very general conclusion that recombination between disparate genomes has great potential to produce novel phenotypes (Gavrilets 1999).

## Predictions

The primary prediction characterizing many years of speciation research is that hybridization between disparate genomes will often generate novel phenotypes that are inviable or sterile (“hopeless monsters”), and this becomes ever more likely with increasing differentiation (Dobzhansky 1937; Mayr 1942; Muller 1942; Orr and Turelli 2001; Coyne and Orr 2004; Gavrilets 2004). At the same time, the number of potentially beneficial interactions might increase (Stelkens and Seehausen 2009; Stelkens et al. 2009), leading to a race between the potential for hybrid speciation and the evolution of complete reproductive isolation. Here, as in the case of mutations of large effect, there is probably an inverse relationship between the magnitude of a transgressive beneficial phenotype and the likelihood that it will actually be generated in nature.

The most important prediction arising from hybridization as a source of novelty is that admixed populations with many recombinant individuals repeatedly bring together many genetic differences in many unique combinations. These two key features can facilitate rapid adaptive evolution of a new phenotype. First, instead of a single genetic difference, the diversity of recombinant genotypes after the F_1_ generation provides a wide field for selection of beneficial versus deleterious interactions (Lexer et al. 2003; Parsons et al. 2011). As pointed out by Arnold and Hodges (1995), this means that even if most hybrid interactions are deleterious, there is still a good chance for the rare beneficial recombinant to appear, unless F_1_ hybrids are completely sterile or inviable. Second, segregating hybrid populations will repeatedly produce recombinant genotypes with transgressive phenotypes (Figs. 2, 3), instead of only producing a single unique mutant or rare variant likely to be lost, even if advantageous (Gillespie 2004). This means hopeful monsters produced by transgressive segregation have a good chance of finding suitably monstrous mates in a hybrid population and can establish a true-breeding population derived from many independent interspecific matings (Bateson 2002).

Although speciation by transgressive hybridization is expected to be rapid in diploids (Ungerer et al. 1998), we predict fixation of novel transgressive hybrids to be more rapid and perhaps more common in haploid and allopolyploid hybrids. All of the recombinant hybrids in haploid and allopolyploid populations will be true-breeding, compared to just a fraction of diploid recombinant hybrids (Fig. 2). In the case of complete or incomplete dominance of *A* and *B*, all four diploid recombinant genotypes will exhibit a transgressive phenotype, but only the double homozygote will be true-breeding. This might lead to lower average fitness of a diploid hybrid population that contains some high-fitness transgressive phenotypes for several generations after hybridization is initiated (Johnson et al. 2010).

Finally, other more subtle predictions might arise from variation in genomic structure and development. For example, the Dobzhansky-Muller model helps explain empirical generalizations including Haldane’s Rule and the large-X effect in hybrid dysfunction. By extension, the expression of beneficial transgressive phenotypes might differ between sex chromosomes and autosomes, with differential consequences for males and females in lineages with chromosomal sex determination. Specifically, if transgressive phenotypes are often recessive (*s*_0_ < ½ *s*_1_ < ½ *s*_2_ in Table 1B) and one or more of the interacting genes is on the sex chromosome, then the phenotype is more likely to be expressed in the heterogametic sex, even in the F_1_ generation. Whether such “rules” might exist for transgressive phenotypes depends largely on whether dominance is a consistent effect in trait expression. The only broad generalization emerging from reviews of the empirical literature so far appears to be that the additive complementation model is often adequate to explain the data (Rieseberg et al. 1999; Burke and Arnold 2001). However, epistasis and dominance are not infrequently detected, and the difference might reflect lower statistical power to detect non-additive effects.

## Conclusions

The idea that hybridization can rapidly produce novel forms is familiar among botanists, but rarely appeared in mainstream discussions of speciation until recently thanks to several case studies of homoploid hybrid speciation (for reviews see: Arnold 1997; Rieseberg et al. 1999; Rieseberg et al. 2003; Arnold 2006; Mallet 2007). Recombination of fixed genetic differences between two populations in the F_2_ and later generations can produce hybrids with phenotypes novel to both parental populations (Fig. 3). When these recombinant phenotypes have fitness beyond the range of parental phenotypes they are transgressive (Fig. 1).

Bateson’s model of hybridogenic hopeful monsters and the Dobzhansky-Muller incompatibility model of hybrid inviability are both cases of transgressive segregation. The Dobzhansky-Muller model produces a “hopeless monster”: hopeless because sterility and inviability make finding a mate and/or novel niche moot and monstrous because sterility and inviability are both phenotypes outside the parental range of phenotypes (Table 1A). The Bateson model produces a hopeful monster: hopeful because it has a good chance of finding a mate given continued hybridization and greater fitness than parental phenotypes in some environments, and monstrous because of its transgressive phenotype (Table 1B). The complementation model can produce both (Table 1C). All three models are special cases of the general quantitative genetic model, thus reconciling sudden and gradual origins of novelty without requiring a special class of mutations or population dynamics.

Transgressive segregation might be an important mechanism promoting sudden phenotypic changes and ecological transitions in evolution. Even if most of the variation produced is deleterious, a rare transgressive hybrid genotype could rapidly fix in a population or establish a novel lineage. It is even possible that regularities in the distribution of dominance effects could lead to general predictions (such as the large X effect and Haldane’s Rule) for transgressive trait expression, but more research on the genetic architecture of transgressive traits is needed. Regardless of those details, admixture can simultaneously bring together many new combinations of alleles, generating multilocus novelties that might never have appeared via gradual accumulation of new mutations in a single population. Gene exchange is not the sole, nor even necessarily most likely, source of evolutionary novelty (Meyer 2002; Moczek 2008), but is perhaps the most likely mechanism of sudden, population level change. Transgressive segregation might be just the mechanism to make more monsters hopeful.
